# lncRNA DLEU2 acts as a miR-181a sponge to regulate SEPP1 and inhibit skeletal muscle differentiation and regeneration

**DOI:** 10.18632/aging.104095

**Published:** 2020-11-18

**Authors:** Yao Wang, Zhi-Jie Zhao, Xue-Ran Kang, Tao Bian, Zhe-Min Shen, Yang Jiang, Bao Sun, Han-Bing Hu, Yi-Sheng Chen

**Affiliations:** 1 Department of Orthopedics, Jinshan Hospital of Fundan University, Shanghai Medical College of Fudan University, Shanghai, China; 2 Department of Surgery, Jinshan Hospital of Fundan University, Department of Orthopedics, Shanghai General Hospital, Shanghai Jiao Tong University School of Medicine, Shanghai, China; 3 Department of Neurosurgery, Shanghai Ninth People’s Hospital, Shanghai JiaoTong University School of Medicine, Shanghai, China; 4 Department of Otolaryngology-Head and Neck Surgery, Shanghai Ninth People’s Hospital, Shanghai Jiao Tong University School of Medicine, Ear Institute, Shanghai JiaoTong University School of Medicine, Shanghai Key Laboratory of Translational Medicine on Ear and Nose diseases, Shanghai, China; 5 Department of Orthopedics, Shanghai General Hospital, Shanghai Jiao Tong University School of Medicine, Shanghai, China; 6 Department of General Surgery, Jinshan Hospital of Fundan University, Shanghai Medical College of Fudan University, Shanghai, China; 7 Department of Neurosurgery, Shanghai General Hospital, Shanghai Jiao Tong University School of Medicine, Shanghai, China; 8 Center of Emergency and Intensive Care Unit, Jinshan Hospital of Fudan University, Shanghai, China

**Keywords:** sarcopenia, competing endogenous RNA (ceRNA), SELENOP protein (SEPP1), miR-181a, DLEU2

## Abstract

Sarcopenia is a serious public health problem associated with the loss of muscle mass and function. The purpose of this study was to identify molecular markers and construct a ceRNA pathway as a significant predictor of sarcopenia. We designed a prediction model to select important differentially expressed mRNAs (DEMs), and constructed a sarcopenia associated ceRNA network. After correlation analysis of each element in the ceRNA network based on clinical samples and GTEX database, C2C12 mouse myoblasts were used as a model to verify the identified ceRNA pathways. A new model for predicting sarcopenia based on four molecular markers SEPP1, SV2A, GOT1, and GFOD1 was developed. The model was used to construct a ceRNA network and showed high accuracy. Correlation analysis showed that the expression levels of lncDLEU2, SEPP1, and miR-181a were closely associated with a high risk of sarcopenia. lncDLEU2 inhibits muscle differentiation and regeneration by acting as a miR-181a sponge regulating SEPP1 expression. In this study, a highly accurate prediction tool was developed to improve the prediction outcomes of sarcopenia. These findings suggest that the lncDLEU2-miR-181a-SEPP1 pathway inhibits muscle differentiation and regeneration. This pathway may be a new therapeutic target for the treatment of sarcopenia.

## INTRODUCTION

Sarcopenia has been reported to be associated with an elevated risk of physical disability and diabetes mellitus. It is characterized by generalized and gradual loss of skeletal muscle mass and strength [1–6]. The prevalence of Sarcopenia increases with age and has become an important factor in the physical health of older people [2, 7, 8]. Skeletal muscle differentiation is controlled by multiple signaling pathways. Myogenic regulation factor (MyoD, MyoG) is the core component of the myogenic pathway. [9] Following the development of sequencing technology, the role of lncRNA as a microRNA sponge to regulate miRNA's ceRNA networks in biological processes has become more widely recognized [10–13]. Several studies have demonstrated that many long non-coding RNAs (lncRNAs), including MAR1, [14] H19, [15, 16] MUMA, [17] Yam-1, [18] IRS1, [19] Malat1, [20] lncR-125b (TCONS_00006810), [21] and lnc-mg, [22] are involved in muscle differentiation and regeneration.

However, previous studies have mainly focused on the pathological role of ceRNA network in sarcopenia, and there are currently no effective methods for assessing the risk of sarcopenia. Therefore, there is a need to develop reliable prediction tools based on identified molecular markers to reduce the risk of sarcopenia.

The purpose of this study was to develop an effective tool for early prediction of the risk of sarcopenia based on several identified molecular markers. *In vitro* experiments revealed that lncRNA DLEU2 as a miR-181a sponge regulates SEPP1 expression and inhibits muscle differentiation and regeneration. This study provides a new therapeutic target for the treatment of age-related sarcopenia.

## RESULTS

### DEMs from GEO datasets

**Figure 1:**
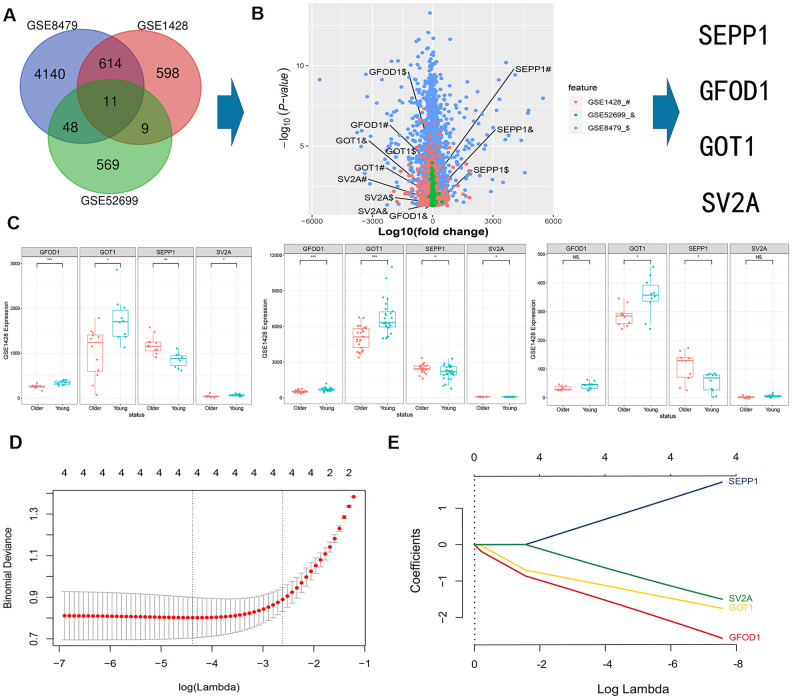
**A Veen diagram showing intersection of mRNAs expression profiles in GSE8479, GSE1428, and GSE52699 datasets.** (**A**) Multi-volcano plot of DEMs in GSE8479, GSE1428, and GSE52699 datasets. (**B, C**) Association between the expression of DEMs (SEPP1, SV2A, GOT1 and GFOD1) in the indicated datasets. * P < 0.05. (**D**) DE-miRNAs selection using LASSO regression model. The selection of the optimal parameters (lambda) in the LASSO model uses the minimum criterion of 5-fold cross-validation. The dashed line was drawn at the best value using the minimum criterion and 1se (standard error) of the minimum criterion. (**E**) LASSO coefficient profiles of the 4 features. Generation of coefficient outline based on the log (lambda) sequence, where the optimal lambda acquires the characteristics of the 4 non-zero coefficients.

The data obtained after normalization revealed that a total of 11 differentially expressed mRNAs were obtained from skeletal muscle samples (GSE8479, GSE1428, and GSE52699) (Figure 1A). Multiple volcano plots of differential expression are presented in Figure 1B. SEPP1 mRNAs were upregulated and GFOD1, GOT1, and SV2A mRNAs were downregulated in the sarcopenia group. The expression levels of mRNAs obtained from the GSE8479, GSE1428 and GSE52699 datasets are shown in Figure 1C.

### DEMs predictors of sarcopenia risk

LASSO regression model was used to select 4 key differentially expressed (SEPP1, GFOD1, GOT1, and SV2A) mRNAs (DEMs) predictors of sarcopenia risk and the lasso parameter (lambda.min) was 0.01038519 (Figure 1D, 1E). The main purpose of using LASSO regression in this study is to prevent the overfitting problem. At the same time, the LASSO regression directly reduces some unnecessary parameters to zero during parameter reduction, further simplifying our model. Thus, this method provides more scientific results. Interestingly, the results of LASSO regression in this study reveals that the four characteristic factors (SEPP1, GOT1, GOFD1, and SV2A) have an important role in the prediction of sarcopenia. Accordingly, these four characteristic factors have not been screened out.

**Figure 2:**
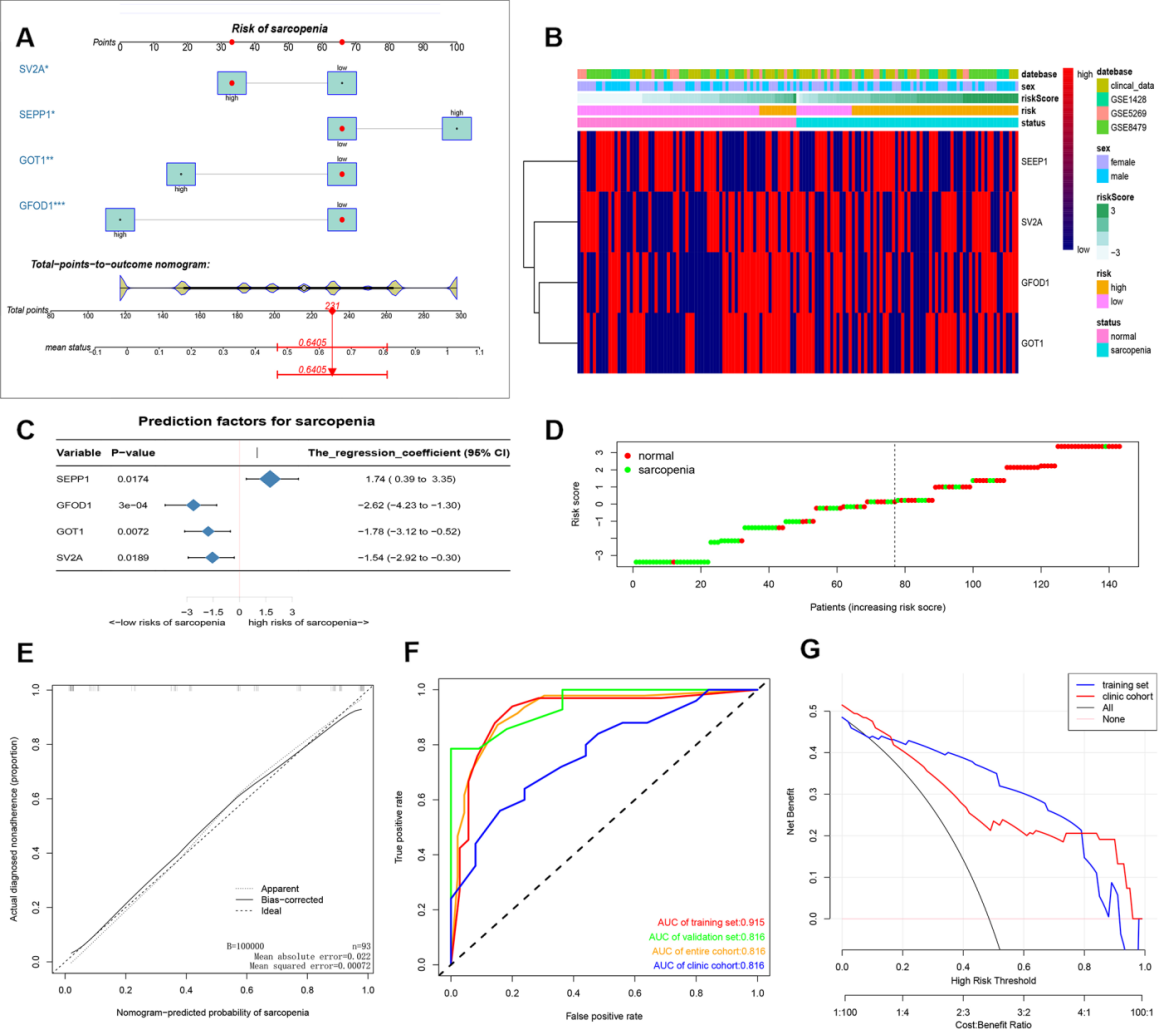
(**A**) prediction nomogram of sarcopenia. Note: expression level of 4 DE-miRNAs, SEPP1, SV2A, GOT1 and GFOD1 are included. (**B**) Heatmap plots of DEMs (SEPP1, SV2A, GOT1 and GFOD1) in clinical samples and GSE8479, GSE1428, and GSE52699 datasets. (**C**) A forest chart showing the prediction factors of sarcopenia. (**D**) Analysis risk scores of sarcopenia based on DEMs in clinical samples and GSE8479, GSE1428, and GSE52699 datasets. (**E**) A calibration curve for the predictive model of sarcopenia. Note: The x-axis is the risk of skeletal muscle reduction whereas the y-axis represents the actual incidence of sarcopenia. Diagonal dashed lines represent perfect predictions for an ideal model. The solid line indicates the prediction ability of the proposed prediction model. The closer the solid line matches the dotted line, the higher the prediction ability. (**F**) The AUC of the nomogram for sarcopenia is equal to the accuracy of randomly selected samples. (**G**) Decision curve showing the benefit probability of the intervention. The figure shows the decision curve for the training set, validation set, the entire cohort and the clinic cohort.

After that, a nomogram model containing the independent predictors is shown in Figure 2A, 2C as well as Supplementary Table 1. In the training cohort, the area under the ROC curve (AUC) was high at 0.915 (Figure 2E, 2F). We also added our clinical data to corroborate our results in Figure 2F and the clinical cohort had an AUC of 0.770. These findings suggested that the nomogram can be used to predict the occurrence of sarcopenia. The C index of the proposed Nomogram was 0.915 (95% CI, 0.840-0.989) which was lower than the validation set 0.945 (95% CI, 0.869-1.000) and higher than the clinical cohort 0.770 (95% CI,0.642-0.897) (Table 1). Besides, we also described accuracy, F-value, precision and recall of each dataset and proposed nomogram in Table 2. These findings suggested a strong discriminatory power and accurate predictive performance. The decision curve analysis of the nomogram is shown in Figure 2G. Therefore, the nomogram is a predictive model in clinical practice that can be used to predict the occurrence of sarcopenia for early and timely intervention. The heat map and risk plot generated for the DEMs and predicted risk score for sarcopenia from the two groups are presented in Figure 2B, 2D.

**Table 1:** C-index of the prediction model.

**Dataset group**	**C-index of the prediction model**	
	**C-index**	**The C-index (95% CI)**
Training set	0.915	0.840-0.989
Validation set	0.945	0.869-1.000
Entire Cohort	0.923	0.866-0.980
Clinic cohort	0.77	0.642-0.897

**Table 2:** Accuracy, F-value, precision and recall of each dataset.

	**Dataset group**			
	**Entire cohort**	**Training set**	**Validation set**	**Clinic cohort**
Accuracy	0.8602	0.8676	0.84	0.68
F-value(α=1)	0.8571	0.8696	0.8181	0.6667
Precision	0.8478	0.8571	0.8181	0.64
Recall	0.8667	0.8824	0.8181	0.6957

Note: The proposed nomogram of accuracy, F-value, precision and recall show in Supplementary Table 2.

### Established ceRNA network

**Figure 3:**
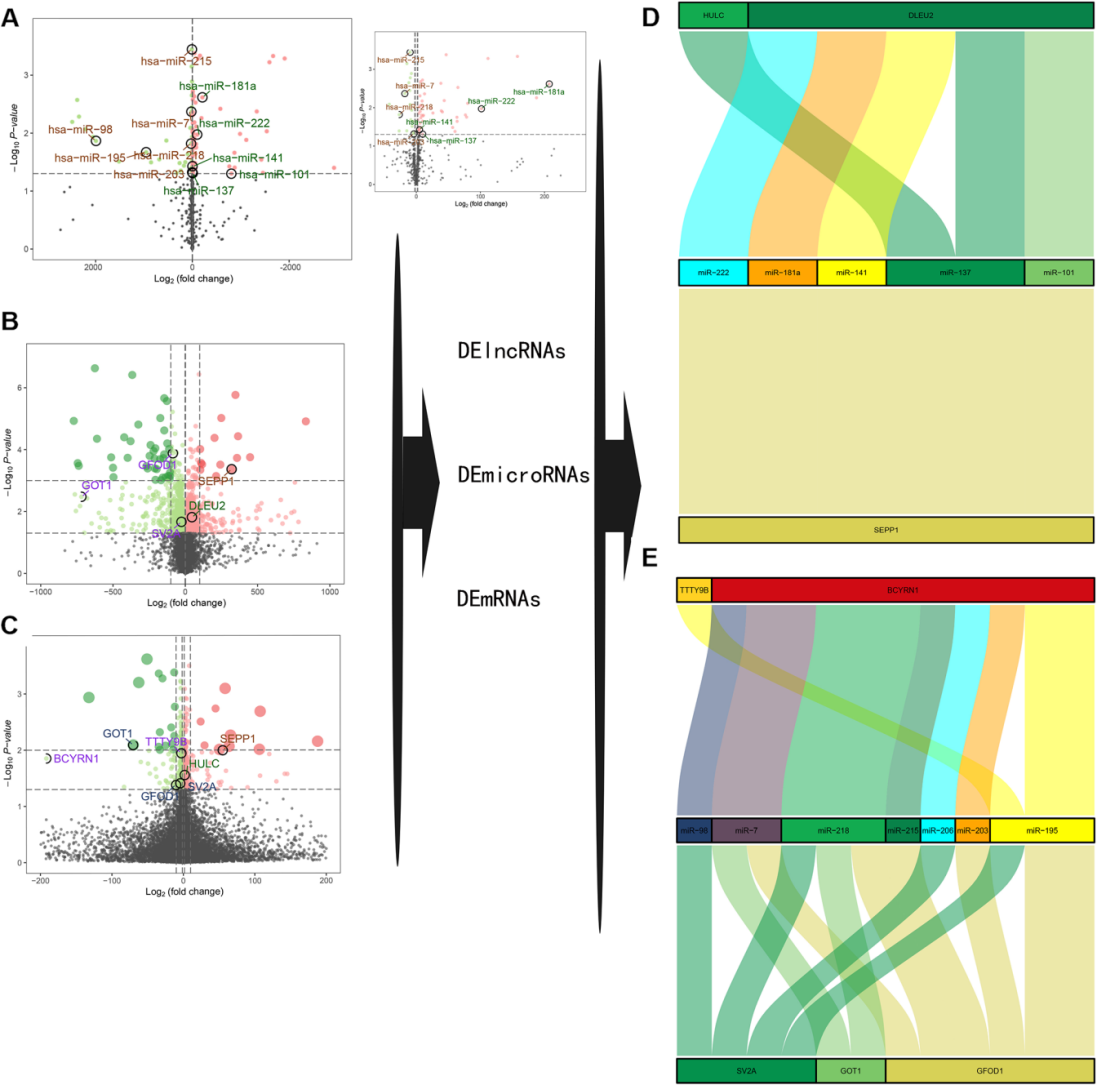
****(**A–C**) A volcano plot of GSE23527 with 886 miRNAs, of which 100 were either up- or down-regulated. (**B**) A volcano plot of GSE1428 with 12427 RNAs; 1232 RNAs and lncRNAs were identified either up- or down-regulated. (**C**) A volcano plot of GSE52699 with 34663 RNAs; 637 RNAs were identified either up- or down-regulated. Note: the red dots represent the upregulated genes, the green dots represent the downregulated genes, and the black dots represent the genes that are not significantly differentially expressed in old muscle samples. (**D**, **E**) A ceRNA network for sarcopenia.

Differentially expressed lncRNAs (DE-lncRNAs) and miRNAs (DE-miRNAs) were obtained by differential expression analysis as previously described. Volcano plots of differential expression are presented in Figure 3A–3C. Construction of a ceRNA network was based on miRcode, miRWalk3.0 and miRTarBase databases as shown in Figure 3D, 3E. Two DE-lncRNAs (TTTY9B and BCYRN1) and five DE-miRNAs (miR-222, miR-181a, miR-141, miR-137, and miR-101) were found to be down-regulated, while two DE-lncRNAs (DLEU2 and HULC) and seven DE-miRNAs (miR-98, miR-7, miR-218, miR-215, miR-206, miR-203, and miR-195) were found to be up-regulated in the sarcopenia group. Combined with the nomogram prediction model results, SEPP1, GFOD1, GOT1, and SV2A may be key modulators of sarcopenia.

Besides, we also describe the correlation between DLEU2, BCYRN1, SEPP1, SV2A, GOT1, and GFOD1 with miR-137 according to the GTEX database in Supplementary Figure 1. The GTEX database is the only skeletal muscle microarray data set that can be collected, while others such as skeletal muscle microarray data sets GSE8479, GSE1428, and GSE52699 do not have a complete information on microRNA transcription or mRNA. Nevertheless, we did not obtain the transcriptome data of lincRNA HULC and lincRNA TTTY98 from the GTEX database, and the expression of most microRNAs (miR-222, -141, -101, -98, -7, -218, -215, -206, -203 and - 195) in skeletal muscle have not been well identified. Therefore, the new correlation analysis can only show the correlation between SEPP1, SV2A, GOT1 and GFOD1, and other lincRNAs (DLEU2, BCYRN1). We previously described the correlation between SEPP1, lncDLEU2, and miR-181a by using clinical data which yielded clear results. Therefore, we should further expand the detection range of our clinical samples (including DLEU2, BCYRN1, HULC, TTTY98, SEPP1, SV2A, GOT1 and GFOD1 and other microRNAs, such as miR-222, -141, -137, -101, -98, -7, -218, -215, -206, -203 and -195) to complete further correlation studies in the future.

### Expression and correlation analysis of DEMs, miR-181a, and DLEU2

**Figure 4:**
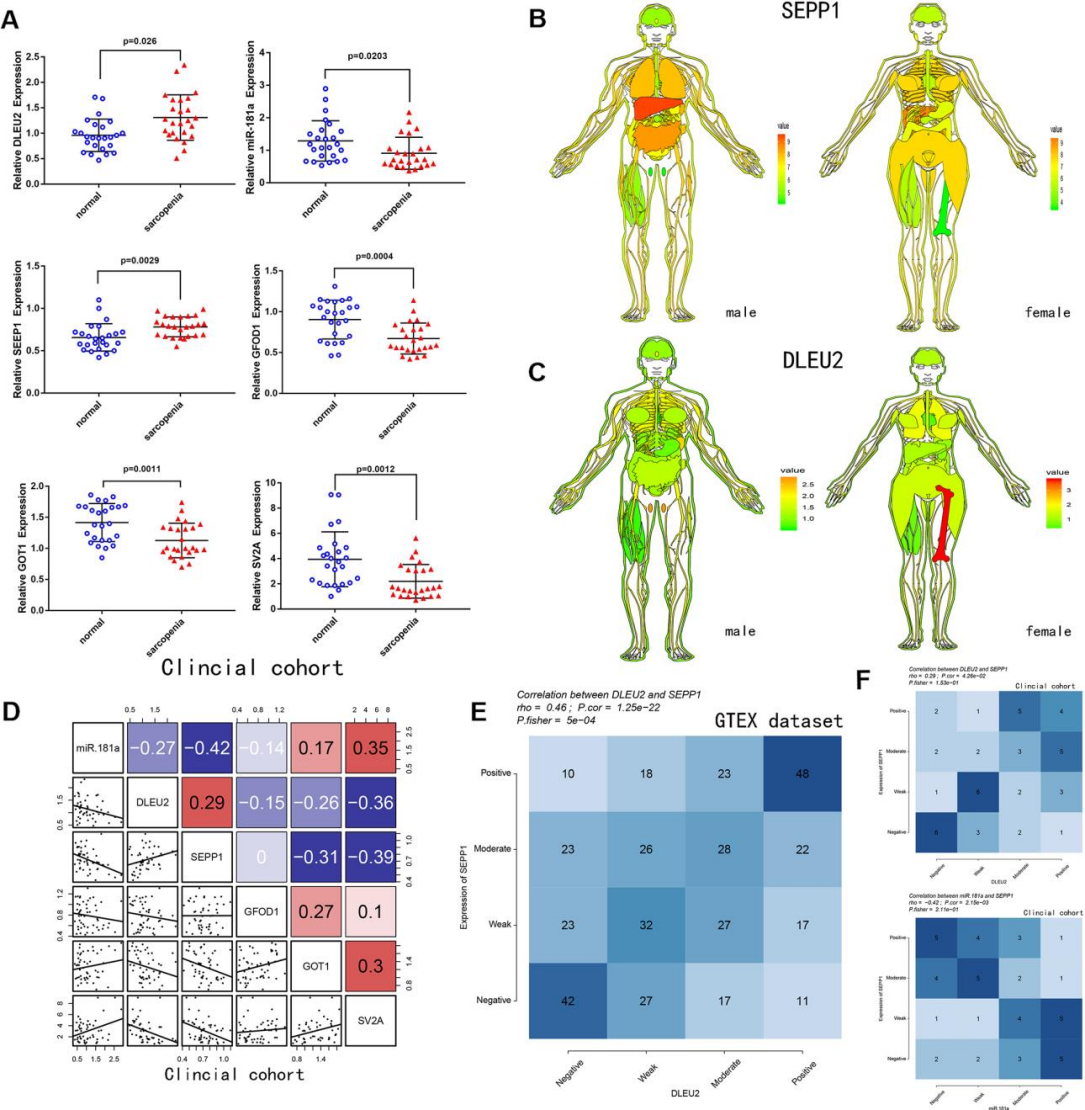
****(**A**) Relative expression of DLEU2, miR-181a, SEPP1, SV2A, GOT1 and GFOD1 as detected by qPCR in samples from clinical cohorts. (**B**) SEPP1 is downregulated in muscle tissues (GTEX cohort; n =7858). An expression map showing SEPP1 protein expression in human tissues. (**C**) DLEU2 is downregulated in muscle tissues (GTEX cohort; n =7858). An expression map showing DLEU2 protein expression in human tissues. (**D**) Correlations among DLEU2, miR-181a, SEPP1, SV2A, GOT1 and GFOD1 in muscle tissues (clinic cohort; n = 50). (**E–F**) Comparison of expression scores of DLEU2 with those of SEPP1 or miR-181a in muscle tissues. The correlations shown are for muscle tissues from clinical cohorts (n=50) and normal muscle tissues from GTEX cohorts (n=396).

A total of 25 patients with sarcopenia and 25 patients without sarcopenia were included in the study. DE- miRNAs (SEPP1, GFOD1, GOT1, and SV2A), miR-181a and DLEU2 expression levels from the patient's muscles were examined using quantitative real-time PCR (Figure 4A). SEPP1 and DLEU2 expression levels were higher in patients with sarcopenia than in patients without sarcopenia, while the expression levels of GFOD1, GOT1, SV2A, and miR-181a were significantly lower in patients with sarcopenia than in patients without sarcopenia.

Correlation and ceRNA network (Figure 3D, 3E) analysis revealed that lncRNA DLEU2 acts as a sponge of miR-181a and up-regulates the expression of SEPP1. Further, in the clinic cohort, correlation analysis showed a significant negative correlation between miR-181a with SEPP1 and DLEU2 (Figure 4D). Besides, there was a significant positive correlation between SEPP1 with DLEU2 both in the GTEX database and clinic cohort (Figure 4E, 4F). To further understand the expression of SEPP1 and DLEU2, human tissue–enriched protein expression maps were constructed. SEPP1 and DLEU2 were found to be lowly enriched in the muscle (Figure 4B, 4C). Therefore, this study revealed that miR-181a could be a protective factor, whereas DLEU2 and SEPP1 could be detrimental to skeletal muscle development.

### DLEU2 inhibited myogenic proliferation and differentiation of C2C12 myoblasts

**Figure 5:**
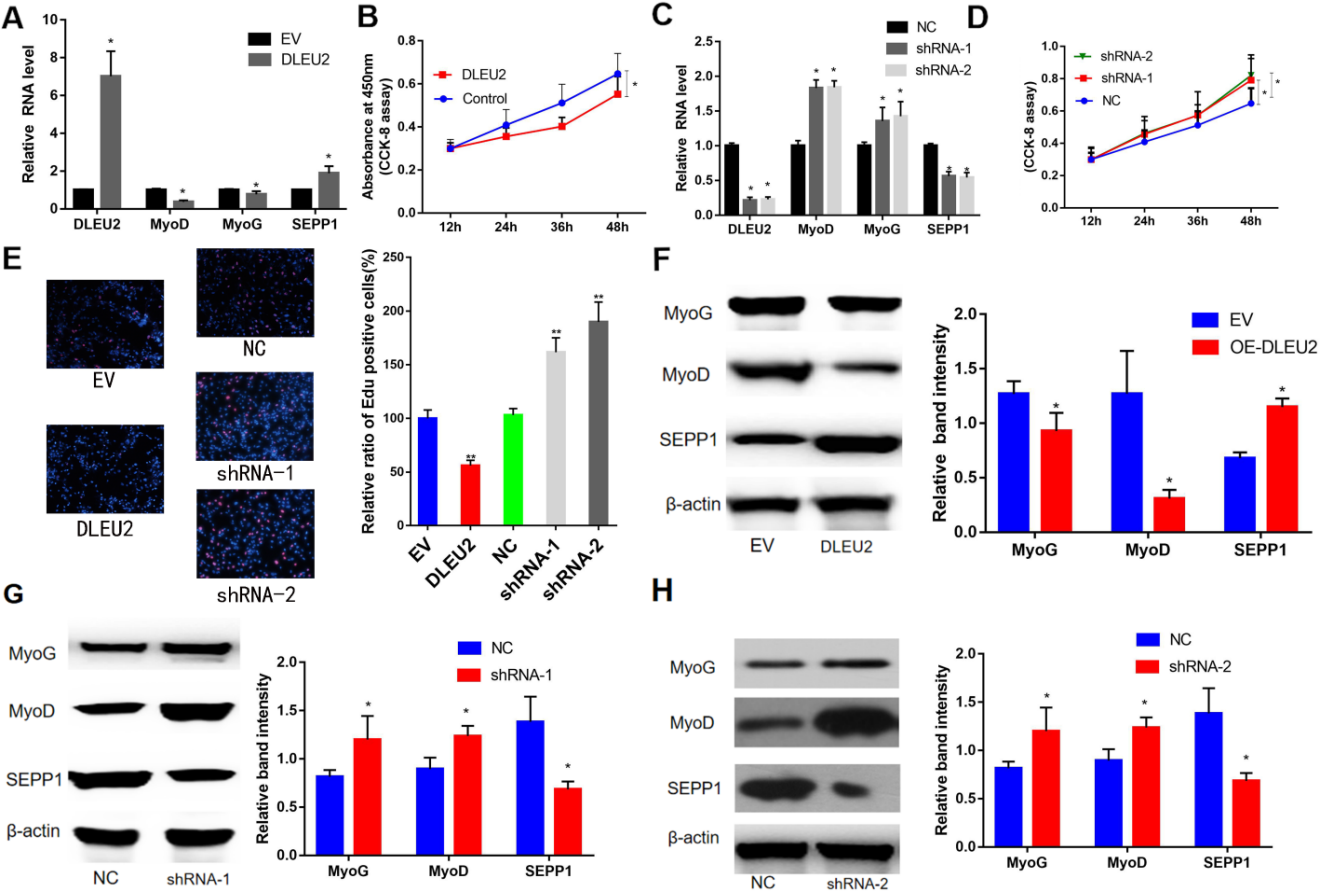
****(**A**) mRNA expression levels of SEPP1 and myogenic markers (MyoD and MyoG) in C2C12 cells transfected with DLEU2 as detected by RT-PCR assay. (**B**) Proliferation of C2C12 cells following DLEU2 overexpression. (**C**) mRNA expression levels of SEPP1, MyoD and MyoG in C2C12 cells transfected with DLEU2 shRNA as detected by RT-PCR assay. (**D**) Proliferation of C2C12 cells following transfection with DLEU2 shRNA. (**E**) C2C12 myoblasts were treated with DLEU2 or shRNA-1/2. EDU assays demonstrated that treatment with DLEU2 reduced cell proliferation and the level of EDU-positive C2C12 cells. Quantification of relative ratio of Edu+ C2C12 cells. Data are presented as the mean ± SD (n = 3). Versus control or NC, ** p < 0.01, ***p <0.005. (**F–H**) Protein expression levels of SEPP1, MyoD and MyoG in C2C12 cells transfected with DLEU2 or DLEU2 shRNA as detected by western blot assay.

In this study, a lentiviral vector encoding with DLEU2 or DLEU2 shRNA was constructed and used to prepare a lentivirus system for C2C12 cells infection. This system was used to demonstrate the functions of DLEU2. The results showed high DLEU2 expression in transduced C2C12 cells (Figure 5A). shDLEU2-1(shRNA-1) showed the highest level of had the highest knockout efficiency in C2C12 cells (Figure 5C). Besides, quantitative RT-PCR analysis revealed a negative correlation between the levels of DLEU2 and differentiation markers of myofibrils (MyoD and MyoG) (P < 0.05, Figure 5A, 5C). CCK-8 and EDU assays demonstrated that treatment with DLEU2 reduced cell proliferation and the level of EDU-positive C2C12 cells (Figure 5B, 5D, 5E). Overexpression of DLEU2 in C2C12 cells significantly reduces the protein and mRNA levels of muscle-derived markers (MyoG and MyoD) and inhibits the proliferation of C2C12 cells; however, overexpression of DLEU2 promoted the protein and mRNA levels of SEPP1, and the shDLEU2 (shRNA-1&2) reduced SEPP1 expression levels in C2C12 cells. (P < 0.05, Figure 5B, 5C, 5F–5H).

### Validation of miR-181a as a DLEU2 target in C2C12 cells

**Figure 6:**
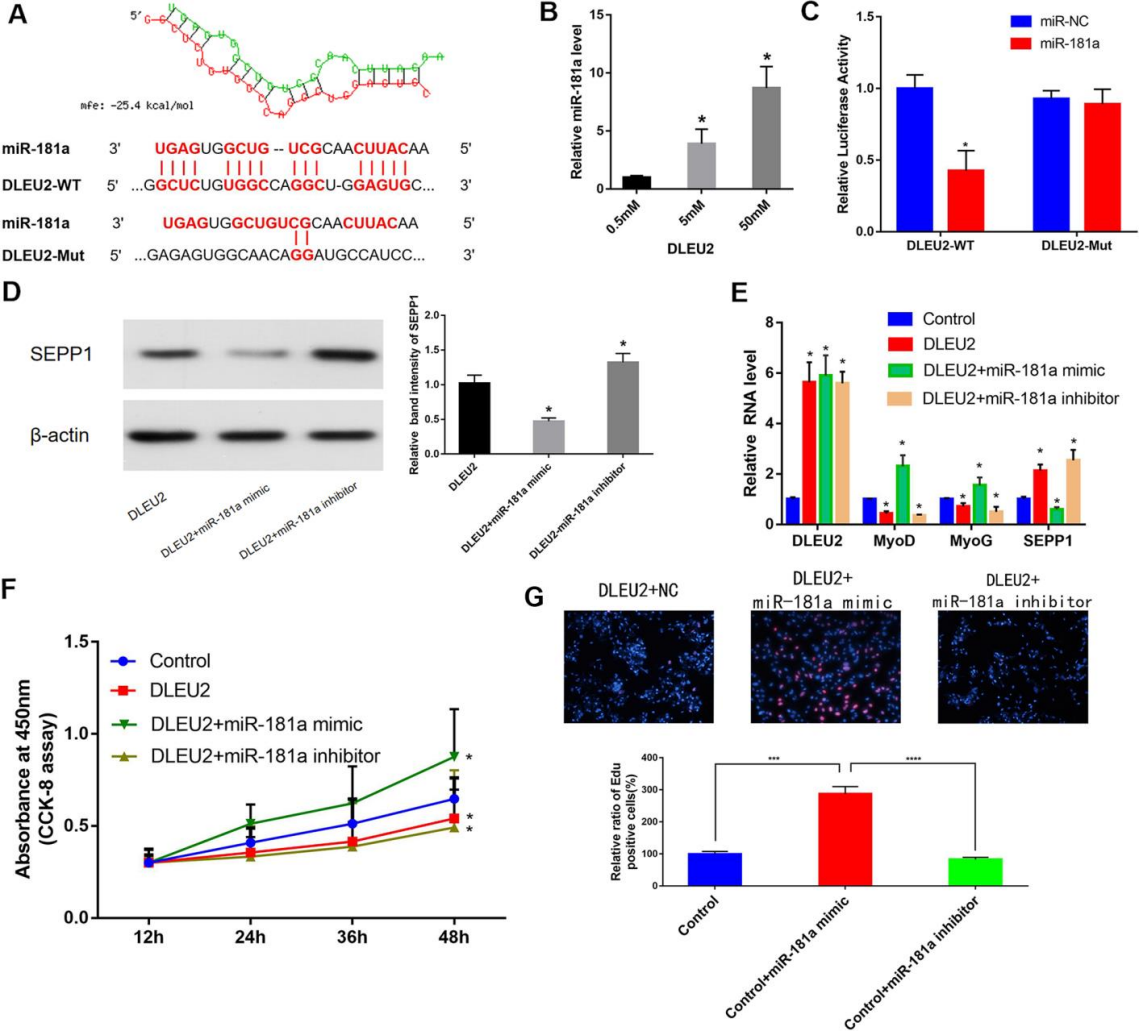
(**A**) Bioinformatics prediction of miR-181a as the target miRNA of DLEU2 using RNAhybrid 2.12. MFE: Minimum free energy. (**B**) C2C12 cells were transfected with different doses of biotin-labeled DLEU2. Results of pull-down experiments for miR-181a and real-time PCR assay results are shown. And biotinylated DLEU2 pulled down some miR-181a in C2C12 cells. * P < 0.05 vs 0.5 mM. (**C**) Determination of miR-181a regulation by DLEU2 by Luciferase reporter assays. * P < 0.05 vs miR-NC. (**D,** **E**) Real-time PCR and Western blot results showing mRNA and protein expression of SEPP1, MyoD and MyoG in C2C12 cells co-transfected with mir-181a mimic or mir-181a inhibitor following DLEU2 overexpression. Data are presented as mean ± SD. U6 small nuclear RNA served as the internal control for lncRNA and miRNA. GAPDH mRNA was used as the control mRNA. (**F**) Effect of DLEU2 overexpression on the proliferation of C2C12 cells. Impact of miR-181a inhibitor and miR-181a mimic of cell proliferation. (**G**) Cells transfected with DLEU2 and treated with the miR-181a inhibitor showed a significant decrease in the level of proliferation, whereas cells treated with the miR-181a mimic showed increased proliferation. Quantification of relative ratio of Edu+ C2C12 cells. Data are shown as the mean ± S.D. (n = 3). *** p < 0.005, **** p < 0.0005.

microRNA181a (miR-181a) was predicted to be one of the target miRNAs of DLEU2 to provide an understanding of the biological mechanism of DLEU2 in regulating muscle differentiation. This statement is based on our previous bioinformatics analysis of the ceRNA network (Figure 3D and Figure 6A). The correlations between DLEU2 and miR-181a were assessed using the miRcode database. [23] Besides, a binding site between miR-181a and DLEU2 was also predicted using RNA hybrid 2.12 (https://bibiserv.cebitec.uni-bielefeld.de/rnahybrid/) (Figure 6A). The miRcode database was used to predict the interactions between DLEU2 and miR-181a [23]. This study revealed that biotinylated DLEU2 pulled down some miR-181a in C2C12 cells (Figure 6B). The double luciferase reporting experiment showed that DLEU2 transfection reduced the luciferase activity of miR-181a, but did not affect the luciferase activity of miR-181a inhibitor. similarly, overexpression of DLEU2 containing the mutant binding site (DLEU2-Mut) did not reduce the luciferase activity of miR-NC and microRNA181a (Figure 6C). Co-transfection with microRNA181a mimic showed increased protein and mRNA levels of muscle-derived markers and decreased levels of SEPP1 in DLEU2 transfected C2C12 cells (Figure 6D, 6E). Besides, EDU and CCK-8 assays examined the proliferation of C2C12 cells after overexpression of DLEU2 and co-transfection with a microRNA181a mimic or microRNA181a inhibitor. Cells transfected with DLEU2 and treated with the miR-181a inhibitor showed a significant decrease in the level of proliferation, whereas cells treated with the miR-181a mimic showed increased proliferation (Figure 6F, 6G).

Therefore, SEPP1 protein expression can be increased by transfecting C2C12 cells with DLEU2. miR-181a mimic decreased SEPP1 protein expression and promoted muscle differentiation in C2C12 cells.

### Functional characterization of the SEPP1 subtypes

**Figure 7:**
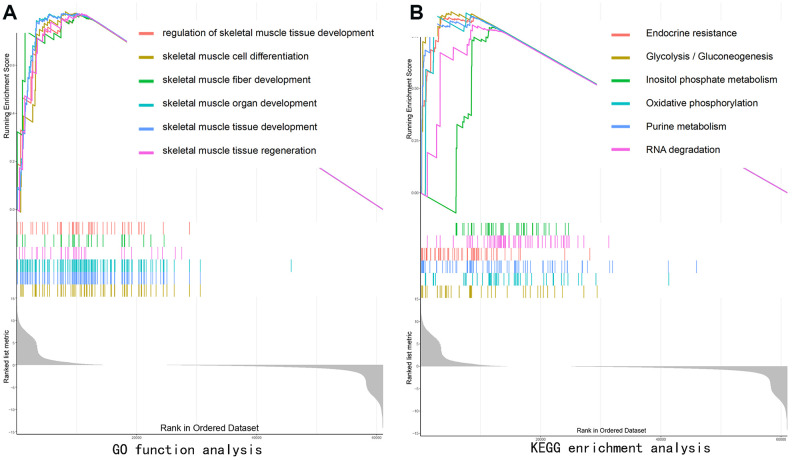
**Gene set enrichment analysis (GSEA) showing the biological pathways and processes associated with SEPP1.** Significant correlations between the high and low SEPP1 expression groups. (**A**) GO enrichment analysis; (**B**) KEGG enrichment analysis.

GO function analysis of GSEA revealed SEPP1-related signaling functions, such as the regulation of skeletal muscle tissue development, skeletal muscle organ development, skeletal muscle fiber development, skeletal muscle cell differentiation, and skeletal muscle tissue regeneration (Figure 7A). KEGG pathways analysis of SEPP1-related pathways revealed the Endocrine resistance pathway, Glycolysis / Gluconeogenesis pathway, Inositol phosphate metabolism pathway, Oxidative phosphorylation pathway, Purine metabolism pathways, and RNA degradation pathways (Figure 7B). These results suggest that SEPP1 plays a vital role in the regeneration and development of muscles and pathways of endocrine resistance and cellular metabolism.

### DLEU2 promotes the expression of SEPP1 protein

**Figure 8:**
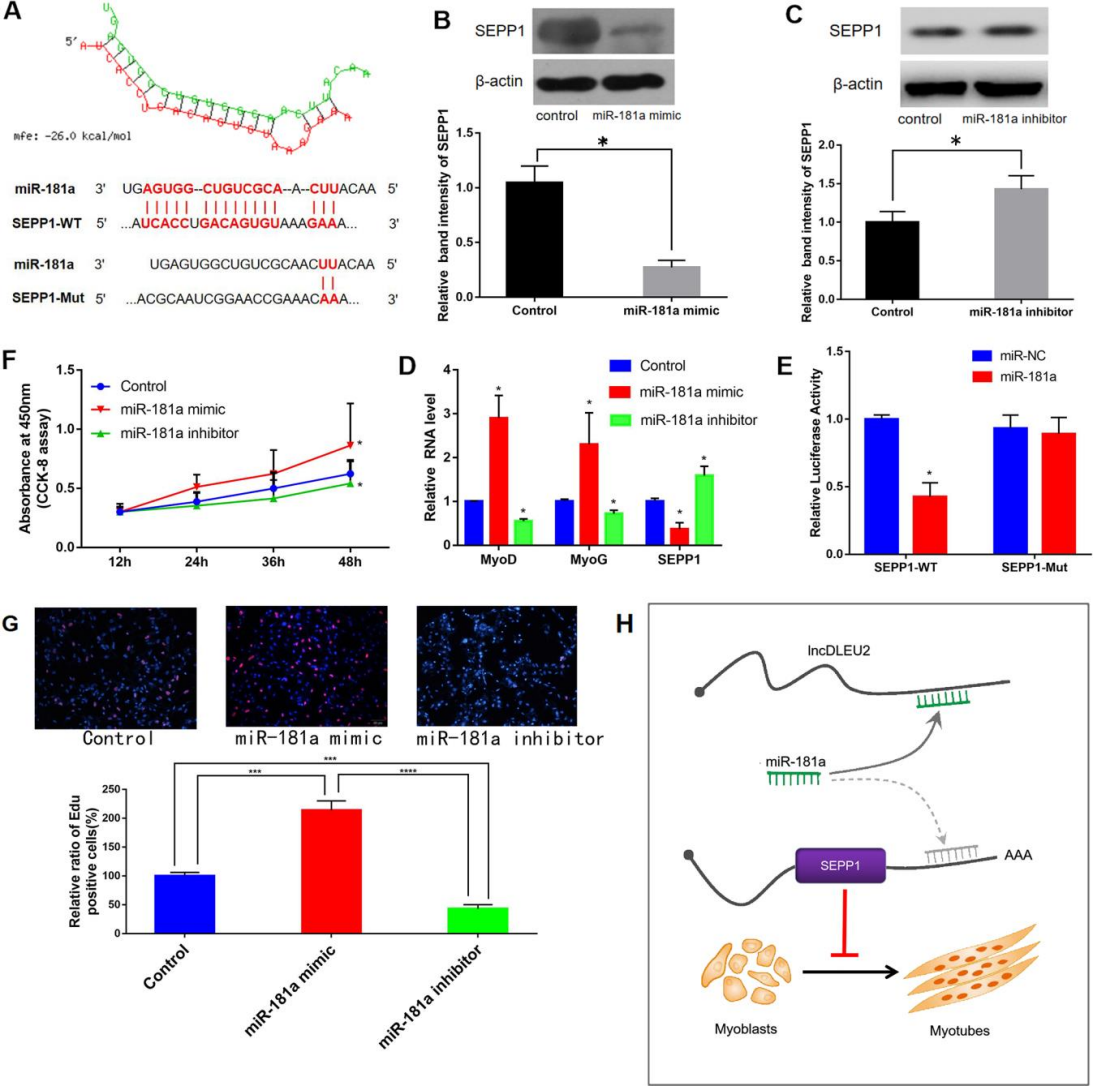
(**A**) Bioinformatic prediction of binding site of miR-181a on DLEU2. (**B, C**) Protein expression of SEPP1 in C2C12 cells transfected with miR-181a inhibitor or miR-181a mimic as determined by Western blot assay. * P < 0.05 vs control, each test was performed in triplicate. (**D**) Gene expression of *SEPP1, MyoD* and *MyoG* in C2C12 cells transfected with miR-181a inhibitor or miR-181a mimic as determined by RT-PCR assay. *P < 0.05 vs control, each test was performed in triplicate. (**E**) Analysis of miR-181a regulation by SEPP1 using Luciferase reporter assays. *P < 0.05 vs miR-NC. (**F**) Effect of DLEU2 overexpression, miR-181a inhibitor and miR-181a mimic on the proliferation of C2C12 cells. (**G**) C2C12 myoblasts were treated with miR-181mimic or miR-181inhibitor. Cells were stained with Edu. Quantification of relative ratio of Edu+ C2C12 cells. Data are presented as the mean ± S.D. (n = 3). ***p<0.005, ****p<0.0005. (**H**) A model showing the inhibitory effect of lncDLEU2-miR-181a-SEPP1 pathway in muscle differentiation and proliferation. LncRNA DLEU2 as a miR-181a sponge regulates SEPP1 expression and inhibits muscle differentiation and regeneration.

MiRWalk and miRcode databases predicted that miR-181a regulated SEPP1 expression (Figure 8A). Luciferase reporter assays revealed that C2C12 cells co-transfected with SEPP1-WT and miR-181a showed lower luciferase activity compared to those co-transfected with SEPP1-WT and microRNA-negative control (NC; p < 0.05) (Figure 8D). Besides, forced expression of miR-181a in C2C12 cells down-regulates SPEE1 protein expression, while miR-181a inhibitor up-regulates SPEE1 expression (Figure 8B, 8C, 8E). Cells treated with the miR-181a mimic exhibited significantly higher proliferation compared with the others (Figure 8F). These results indicated that miR-181a is a key regulator of SEPP1 expression in muscle. In conclusion, lncRNA DLEU2 acts as a miR-181a sponge to regulate the expression of SEPP1, thereby promoting muscle proliferation and differentiation. (Figure 8H).

## DISCUSSION

In this study, lncRNA DLEU2 was found to act as a miR-181a sponge and inhibited skeletal muscle regeneration and differentiation. The lncDLEU2-miR-181a-SEPP1 pathway inhibits muscle differentiation and regeneration and can be used as a novel therapeutic target for the treatment of age-associated sarcopenia. Besides, this study is the first to predict the occurrence of sarcopenia based on four molecular markers (SEPP1, SV2A, GOT1, and GFOD1). The prediction model showed high accuracy based on the detection of clinical samples, hence it can be used to evaluate clinical effectiveness and prognosis [24].

Several studies have demonstrated the important role of the ceRNA network in sarcopenia and emphasized the need to systematically identify altered mRNAs, miRNAs, and lncRNAs in skeletal muscle development [10, 14–16, 18–22]. In this study, a sarcopenia ceRNA network was constructed by combining bioinformatics analysis and sarcopenia prediction models. The prediction model was based on four molecular markers and gave a high C index and AUC value after validation of clinical data. This indicated that the prediction model can accurately assess the risk of sarcopenia. Analysis of clinical sample data and correlation analysis of GTEX database selected the lncDLEU2-miR-181a-SEPP1 pathway as a potential target for further research. *In vitro* cell experiments confirmed that DLEU2 is a miR-181a sponge that up-regulated SEPP1 expression and inhibited muscle proliferation and differentiation in C2C12 cells.

DLEU2 gene is conserved in humans and mice [25]. It is reported to act as a microRNA sponge (miR-455, miR-496, miR-30c-5p, etc.) and can block cell proliferation in several ways [19, 26–29]. DLEU2 is highly expressed in patients with osteoporosis [30]. Healthy skeletal muscles are required for the prevention of osteoporosis [31]. In this study, high expression of DLEU2 was considered to be one of the risk factors for sarcopenia in older people. *In vitro* cell experiments showed that lncDLEU2 silencing promoted the differentiation and proliferation of C2C12 cells, while forced lncDLEU2 expression inhibited the proliferation and differentiation of C2C12 cells. These results show that DLEU2 is a negative regulator of skeletal muscle development.

In this study, through bioinformatics analysis, we predicted that miR-181a contains a DLEU2 binding site. The luciferase reporter assay and pull-down assay confirmed the direct binding of miR-181a's response elements to the DLEU2 transcription. Current research shows that miR-181a is important in the establishment of muscle phenotype and is significantly expressed during skeletal muscle cell differentiation [32]. MiR-181 inhibits the occurrence of sarcopenia mainly through 2 ways: (1) miR-181 increases the expression of MyoD and MyoG and promotes myogenic differentiation and expression of muscle markers [33, 34]; (2) It increase the proportion of type II muscle fibers in skeletal muscle, thereby increasing skeletal muscle strength [35]. This study also found that lncDLEU2 interacts with miR-181a to reduce MyoD and MyoG after transcription.

The above findings show that microRNA181a plays a protective role in the differentiation and proliferation of skeletal muscle. In this study, the luciferase assay confirmed the binding of microRNA181a as well as SEPP1 in C2C12 cells. Further, *in vitro* studies revealed that miR-181a may promote muscle cell proliferation and differentiation through targeted down-regulation of SEPP1 protein. SEPP1 may inhibit muscle cell proliferation and differentiation through multiple pathways. First, SEPP1 inhibits oxidative phosphorylation (activation) of key mediators in energy metabolism [36], this may be associated with the pathogenesis of sarcopenia. SEPP1 may be detrimental to the growth and development of muscle cells since studies have reported that the expression of SEPP1 in muscle is relatively low compared to other tissues, such as the brain and testes, and these findings are consistent with those reported in this study [37]. Second, researchers have suggested that the high expression of SEPP1 may lead to selenium deficiency [38]. Selenium deficiency induces muscular dystrophy [38] Numerous studies have highlighted a bidirectional relationship between sarcopenia and diabetes mellitus [39–41]. Interestingly, abnormal glucose metabolism in diabetic patients also leads to upregulated Sepp1 expression [36], and high expression of SEPP1 may be one of the mechanisms through which diabetes causes sarcopenia.

SEPP1 has been reported to be positively correlated with TNF-α levels, and high expression of TNF-α is characteristic of primary muscle disease and high glucose microenvironment [42, 43]. In summary, DLEU2 knockout or over-expression in C2C12 cells results in upregulation or downregulation of the expression level of miR-181a, respectively. These results in a decrease or increase in the level of SEPP1 protein, and ultimately in up or down-regulation of muscle proliferation and differentiation, respectively. These data suggest that DLEU2 may interact with miR-181a to up-regulate the level of SEPP1 protein after transcription.

The muscle mass and strength tend to decrease with increased age. The prevalence of sarcopenia in older adults aged 60-70 is 13%, and it increases to 50% for those aged 80 and above [2, 8, 44]. Accurate risk assessment allows health care providers to assess the risk or likelihood of future occurrence of illness and timely interventions. Therefore, in this study, we developed an effective prediction model for sarcopenia, which can be used to guide the treatment of sarcopenia. This was achieved by first establishing a ceRNA network based on the clinical prediction model and verification by *in vitro* experiments.

However, this study also had some limitations. First, due to the limitations of the data sources, future studies will require that the prediction model includes more factors and increase the sample size. Second, this study only included the most significantly DE-lncRNAs, DE-miRNAs and DEMs in the analysis and ceRNA network construction. Third, the verification of the ceRNA network requires further animal experiments.

In summary, this study found that lncRNA lncDLEU2 acts as a miR-181a sponge to regulate the SEPP1 protein expression, thereby inhibiting muscle proliferation and differentiation. This may be a new therapeutic target for reversing aging skeletal muscle atrophy. A new prediction model with high accuracy can be developed based on four identified molecular markers (SEPP1, SV2A, GOT1, and GFOD1) and used by clinicians to predict the risk of sarcopenia.

## MATERIALS AND METHODS

### Study participants

Patients who underwent patellar surgery at the Shanghai First People’s Hospital between January 2013 and October 2018 were identified and recruited to participate in this study. Patients data were collected through telephone interviews, outpatient services, and community follow-up. Our objective was to develop a novel model to predict the risk of sarcopenia. According to our previous research, many patients suffer from sarcopenia between the ages of 55 and 60 years, so patients aged 55 or older was included in this study. [45] Therefore, fracture patients aged ≥55 years were included. The laboratory muscle tissue test results considered were those obtained from fine-needle aspiration biopsy of the quadriceps femoris muscle. Written informed consent was sought from all the enrolled subjects. The study was conducted strictly under the guidelines as well as regulations of the Declaration of Helsinki and was approved by the Institutional Ethics Review Board of Shanghai General Hospital (no. 2019SQ059).

The inclusion criteria were as follows: (1) availability of complete data for baseline clinical characteristics (age, body mass index, etc.) and follow-ups, (2) the patient can understand and make correct and right feedback to the doctor's questions, thus the doctor collects the correct basic information of the patient, (3) age ≥55 years, (4) diagnosis of sarcopenia according to AWGS criteria, (5) patients managed with self-care before surgery.

A total of Seventy-five patients were screened, out of which fifty patients met the inclusion criteria, completed the questionnaire and the laboratory muscle tissue test. The patients included 24 males (age: 55–88 years, mean age: 70.3 ± 9.1years) and 26 females (age: 59–86 years, average age: 68.3 ± 7.3 years) (Table 3).

**Table 3:** Differences between demographic and clinical characteristics of sarcopenia and normal groups.

**Characteristics**	**Sarcopenia (n=**25)	**Normal (n=25)**	**Total (n=**50)	**P value**
**Gender**				**P>0.05**
Female	12(48%)	14(56%)	26(52%)	
Male	13(52%)	11(44%)	24(48%)	
Age				P<0.01
Mean (SD)	72.0(9.1)	66.5(6.3)	69.3(8.2)	
Median [MIN, MAX]	71[55,]	66[58,80]	68.5[55,88]	
Education_level				P>0.05
Primary (0–6 years)	8(32%)	5(20%)	13(26%)	
Secondary (7–12 years)	13(52%)	14(56%)	27(54%)	
Higher (>12 years)	4(16%)	6(24%)	10(20%)	
Weight				P<0.01
Mean (SD)	57.6(6.3)	63.6(7.7)	60.6(7.6)	
Median [MIN, MAX]	58[43,]	66[48,78]	60[43,67]	
Height				P>0.05
Mean (SD)	163.1(11.7)	163.8(8.4)	163.4(10.1)	
Median [MIN, MAX]	161[140,]	164[151,182]	162.5[140,185]	
BMI				P<0.05
Mean (SD)	21.7(2.5)	23.8(3.2)	15(30%)	
Median [MIN, MAX]	21.4[17.5,]	24.3[17.4,30.2]	35(70%)	
Drinking				P>0.05
Yes	5(20%)	10(40%)	15(30%)	
No	20(80%)	15(60%)	35(70%)	
Smoking				P>0.05
Yes	10(40%)	5(20%)	15(30%)	
No	15(60%)	20(80%)	35(70%)	
Exercise				P>0.05
>2h/week	13(52%)	20(80%)	33(66%)	
≤2h/week	12(48%)	5(20%)	17(34%)	
Surgical technique				P>0.05
Cannulated screws+Standard tension band	25(100%)	25(100%)	50(100%)	
Rehabilitation training				P>0.05
Yes	18(72%)	18(72%)	36(72%)	
No	7(28%)	7(28%)	14(28%)	
Internal fixation removal				P>0.05
Yes	7(28%)	7(28%)	14(28%)	
No	18(72%)	18(72%)	36(72%)	
Heart disease				P>0.05
Yes	4(16%)	1(4%)	5(10%)	
No	21(84%)	24(96%)	45(90%)	
Diabetes				P>0.05
Yes	15(60%)	8(32%)	15(30%)	
No	10(40%)	17(68%)	35(70%)	
High blood pressure				P>0.05
Yes	10(40%)	5(20%)	15(30%)	
No	15(60%)	20(80%)	35(70%)	
Hyperlipidemia				P>0.05
Yes	5(20%)	6(24%)	11(22%)	
No	20(80%)	19(76%)	39(78%)	
Blood type				P>0.05
A	9(36%)	12(48%)	21(42%)	
B	2(8%)	1(4%)	3(6%)	
AB	6(24%)	6(24%)	12(24%)	
O	8(32%)	6(24%)	0(0%)	
Perioperative blood transfusion				P>0.05
Yes	0(0%)	0(0%)	0(0%)	
No	25(100%)	25(100%)	50(100%)	
Follow-up time(year)				P>0.05
Mean (SD)	3.4(1.5)	3.6(1.5)	3.5(1.5)	
Median [MIN, MAX]	3.25[0.8,]	3.3[0.8,6.3]	3.3[0.8,6.25]	
Length of hospital stay				P>0.05
Mean (SD)	10(3.4)	9.4(4.3)	9.7(3.9)	
Median [MIN, MAX]	10[3,]	9[4,25]	9[3,18]	
Treatment group				P>0.05
Group A	11(44%)	16(64%)	27(54%)	
Group B	14(56%)	9(36%)	23(46%)	
Classification of fracture				P>0.05
Upper or lower pole	10(40%)	8(32%)	18(36%)	
Comminuted fracture	6(24%)	3(12%)	9(18%)	
Transverse	9(36%)	13(52%)	22(44%)	
Vertical fracture	0(0%)	1(4%)	0(0%)	

### Methods of assessment

Similarly to our previous researches [45], the diagnostic criteria for sarcopenia established by the 2014 Asian Working Group for Sarcopenia (AWGS) and EWGSOP2 were used to define sarcopenia [46–49]. And Bioelectrical impedance analysis (BIA) was used to assess muscle mass (Bioimpedance meter, TANITA RD-953, Japan) The BIA results were very similar to those of double-energy X-ray absorptiometry and magnetic resonance imaging. BIA also offers the advantages of safety, technical simplicity, low cost, and high patient compliance [50, 51]. All the results of BIA were standardized using cross-validated Sergi.

### Methods of patellar surgery

All patients with patella fractures included were treated with hollow lag screws and tension band wire internal fixation, a commonly used method of patella fracture surgical reduction in our clinical work. We have added the surgical method and the specific operation as follows: Under general anesthesia, a straight or S-shaped incision was made in the front of the knee. Next, the patella was fully exposed and accurately reset under direct vision, then fixed with a two point-shaped reduction forceps to ensure a flat joint surface. Afterward, a special guide needle was used to insert the needle from the edge of the patella slightly behind the central axis of the patella to the opposite edge. Notably, the two needles must be perpendicular to the fracture line, and the needle distance is about 1.5 to 2.0 cm. The X-ray fluoroscopy showed that the articular surface is flat and the position of the guide pin was correct and a hollow drill was used to drill the hole. A Φ3.mm hollow tension nail of appropriate length was selected and screwed it into the patella along the guide pin. Afterward, the two nails were pressurized simultaneously to be firm. The point-shaped reduction forceps were removed and the guide needle was withdrawn. Thereafter, the hollow nail with a Φ0.8mm steel wire was passed and fixed in a "U" shape in front of the patella according to the tension band method.

### Data retrieval

The dataset used in this study was retrieved from the GEO database (http://www.ncbi.nlm.nih.gov/geo/). mRNA or miRNA expression datasets of muscle from humans with sarcopenia were included. The GSE23527 miRNA expression array dataset with relatively high data quality and large sample size, based on the GPL10358 platform (LC_MRA-1001_miRHuman_11.0_080411 (miRNA ID version)), was selected. Datasets containing microarray data from human muscle samples exhibiting sarcopenia and normal were also selected based on three datasets (GSE8479-GPL2700, Sentrix HumanRef-8 Expression BeadChip; GSE1428-GPL96, [HG-U133A] Affymetrix Human Genome U133A Array; and GSE52699-GPL10558, Illumina HumanHT-12 V4.0 expression beadchip). All the original platform files were saved.

### Identification of differentially expressed genes

All data were normalized using the “normalize between array” function of the “LIMMA” R package from the Bioconductor project [52]. This package was also used to identify differentially expressed lncRNAs (DE-lncRNAs), mRNAs (DEMs), and miRNAs (DE-miRNAs) between sarcopenia and normal samples from the GSE23527, GSE8479, GSE1428 and GSE52699 datasets [53]. Threshold for statistical significance was set at P < 0.05 and |logFC| > 1.

### Logistic regression model of risk of sarcopenia

The series matrix files from the GEO datasets (GSE8479, GSE1428, and GSE52699) were downloaded. Skeletal muscle data in the sarcopenia (N = 47) and normal (N = 46) groups were analyzed using R software (version 3.5.3). The samples were randomly selected into the training and validation (7: 3) groups by using the "caret" package. In our research, the "caret" package was only used to divide the samples into the training and validation (7: 3) groups which seed (hyperparameter) was set as 2000. [54] Analyses were performed to identify and evaluate the models. The resulting model was verified using clinical data (25 sarcopenia, 25 normal). All results were saved in text format for subsequent hierarchical clustering analysis using the Complex Heatmap package.

LASSO regression model, which is used widely to reduce high-dimensional data, was used to identify relevant risk factors in sarcopenia [55–57]. The LASSO regression is similar to ridge regression. The reason why we use LASSO regression is that LASSO regression can be understood by adding an L1 regular term based on linear regression. And the LASSO has a certain feature selection function since it uses the L1 regular term. This is because the L1 regular can compress to 0 the coefficients corresponding to some "useless for tags", and subsequently highlight the features that have a better impact on the result. The L2 regular term in the ridge regression does not have this function. It will only state that the coefficients of some irrelevant features are reduced to a smaller value but not reduced to 0. In summary, compared with ridge regression, LASSO regression has more advantages in variable selection and has been extensively used in variable selection. Considering the actual value of predictive models in clinical applications, we consider using as few feature factors as possible to build a more accurate predictive model. Our main purpose in this step is to filter the core variables. [44, 58] Therefore, we selected LASSO regression to filter variables instead of ridge regression. Multivariate logistic regression analysis was then used to establish a predictive model which included the selected features (two-sided P < 0.05) [59]. Odds ratios and 95% confidence intervals (CIs) were calculated after that.

A predictive model was established for predicting sarcopenia risk based on all potential predictors of sarcopenia [60, 61]. A nomogram was established to predict the risk of sarcopenia. Calibration accuracy was statistically assessed using the “rms” package, and high significance indicated that the model could provide accurate calibration [62]. The C-index was calculated to assess the model performance and the biased performance of the sarcopenia nomogram was corrected by bootstrapping (1,000,000 bootstrap resampling) [62]. Besides, we also described accuracy, F-value, precision and recall of each dataset and proposed nomogram as methods show in Supplementary Table 2. The decision curve analysis was also used to assess the clinical usefulness of the nomogram [63]. After that, the net benefit was calculated as the previous study [58].

### Constructing the ceRNA network

A visual co-expression ceRNA network of DE-lncRNAs, DE-miRNAs, and DEMs was constructed using the ggalluvial R software package (version:0.9.1) [64]. The miRcode database was used to confirm the interactions between DE-lncRNAs and DE-miRNAs [23]. The correlations between DEMs and DE-miRNAs were assessed using the miRWalk3.0 database (http://mirwalk.umm.uni-heidelberg.de/), which includes 10 databases (Targetscan, RNA22, PITA, PICTAR5, PICTAR4, RNAhybrid, miRWalk, miRDB, miRanda, and DIANAmT), and the miRTarBase (Version 7.0), which comprises validated miRNA target interactions from experiments [65].

### Data analysis from the GTEX databases

To clarify the correlation between the expression of DE-lncRNAs and DEMs in ceRNA, R software (https://www.r-project.org/) was used to statistically analyze data from the GTEX databases. A human tissue–enriched protein expression map and a boxplot of genes were generated using the “gganatogram” and “ggpubr” models, respectively. Fisher’s exact test or χ² test (two-sided) was used for genotypic correlation analysis.

### Quantitative real-time PCR (qPCR)

Total mRNA and lncRNA was isolated from the cell cultures using the Mini-BEST Universal RNA Extraction kit (TaKaRa, Kyoto, Japan), followed by cDNA synthesis using the Prime-Script RT Master Mix (TaKaRa). qPCR assays were detected using the SYBR Green Master Mix (TaKaRa) with PCR LightCycler480 (Roche Diagnostics, Basel, Switzerland).

Total miRNA was isolated from the cell cultures using TRIzol® reagent (Gibco/Life 270 Technologies, Thermo Fisher Scientific). miRNA quantity and quality were detected by the stem-loop quantitative RT-PCR (TaqMan probe method). Purified miRNA was used for first-strand cDNA synthesis using M-MLV reverse transcriptase and primers according to the manufacturer’s instructions (Promega, Fitchberg, MA, USA). The primer sequences were designed by Primer Premier and the sequences were as follows: microRNA 181a forward 5'-TGAACATTCAACGCTGTCG-3' and reverse 5'-GCAGGGTCCGAGGTATTC-3'.

### Western blot analysis

To determine protein expression, cells were harvested in RIPA buffer containing a protease inhibitor cocktail, and total protein was quantified using a bicinchoninic acid kit (Pierce, Rockford, IL, USA). Aliquots containing 8 μg total protein were separated by sodium dodecyl sulphate polyacrylamide gel electrophoresis and then electro-blotted onto a 0.45-μm PV membrane (Immobilon™; Merck Millipore, Darmstadt, Germany). The membranes were blocked and probed overnight with the primary antibodies anti-SEPP1 (1:1000, #ab193193; Abcam, USA), anti-MyoD (1: 1500; Invitrogen, Carlsbad, CA, USA), anti-MyoG (1: 1500; Invitrogen, Carlsbad, CA, USA), anti–β-catenin (1:5000, #ab32572; Abcam, USA), and anti–active β-catenin (1:500, #05-665; Merck Millipore).

### Differentiation in cell cultures

C2C12 cells were obtained from American Type Culture Collection (ATCC, CRL-1772™, Manassas, VA, USA). The cells were cultured in growth medium consisting of Dulbecco's modified eagle medium (DME-M), 10% heat-inactivated fetal calf serum (Biowest, St. Louis, MO), and 1% penicillin-streptomycin. C2C12 cells were successfully differentiated into myocytes or myotubes in a differentiation medium consisting of DMEM, 2% heat-inactivated horse serum (Invitrogen, Carlsbad, CA, USA) and 1% penicillin-streptomycin. All these cells were maintained at 37° C in a humidified atmosphere containing 5% CO2 [66].

### Cell transfection

This study followed our previously reported protocol with slight modifications. [67] C2C12 cells were cultured to 60% confluence, the culture medium was removed and 1.5×10^8^ IU virus particles added with 8 g/mL hexadimethyl bromide (Sigma-Aldrich, St. Louis, MO, USA). DMEM plus virus particles were changed to DMEM with 10% fiber channel standard and the cells cultured for 1-7 days.

### Construction of lentiviral vectors

To construct the lncDLEU2 overexpression lentiviral vector, we subcloned DLEU2 and the full-length lncDLEU2 into the lentiviral GV112 vector according to the manufacturer's instructions [68]. The vector was provided by Shanghai Genechem (Shanghai, China). For the lncDLEU2-KD lentiviral vector, the shRNA sub-clone of the lncDLEU2 or negative control scramble sequence was used in the GV112 carrier. Shanghai Genechem designed the two shRNA sequences (shDLEU2-1: 5’-AGCTCAGATTCTCTCCTTT-3’, shDLEU2-2: 5’- TGAAAGGTGTACTGCAAGGAA-3’). The lentivirus expression vector was co-transfected into C2C12 cells using TransIT-LT1 (Mirus Bio). The supernatants were collected at 48h and 72h after transfection, concentrated by ultracentrifugation at 25,000 rpm for 90 minutes, and recovered in an appropriate volume of OptiMEM (Gibco, Waltham, MA, USA). Real-time qPCR was used to determine the rapid titer (IU / mL) of infectious virus particles [69].

### Transfection of miRNAs

The transfection of miRNAs was performed as previously described [70]. miR-181a was enhanced and inhibited using chemically synthesized miRNAs mimics and inhibitors (Gene Pharma (Shanghai, China). Cells were seeded and transfection performed using a riboFECT™ CP transfection kit for 24 hours according to the manufacturer's protocol (Ribobio, Guangzhou, China). Real-time quantitative PCR was used to measure the transfection efficiency 48 hours after transfection.

### Pulldown of the biotin-labeled lncDLEU2

Biotin-labeled lncDLEU2 was synthesized by Sangon Biotech (Shanghai, China). Different doses of biotin-labeled DLEU2 (0.5 mM, 5 mM, and 50 mM) were incubated with the cytoplasmic lysate of C2C12 cells transfected with miR-181a at room temperature for 30 minutes. The complexes were isolated using streptavidin-coated magnetic bloodworm (Dynal, Waltham, MA, USA). The captured RNA was purified, washed and subjected to real-time PCR analysis [71].

### Dual-luciferase reporter assay

The putative sequence of the miR-181a binding site and the mutant sequence was cloned into the pmirGlO dual-luciferase miRNA target expression vector (Promega, Madison, WI, USA) to form a reporter vector. Co-transfecting the reporter vector with lncDLEU2- Mut or lncDLEU2-WT into C2C12 cells was performed using Lipofectamine 2000 (Invitrogen, Carlsbad, CA, USA) and similar steps were followed when co-transfecting the reporter vector with SEPP1-WT or SEPP1-Mut. The dual-luciferase reporter assay system (Promega, Madison, WI, USA) was used to detect renilla luciferase activity after 48h, according to the manufacturer’s instructions.

### EDU proliferation and Cell Counting Kit-8 (CCK-8) assay

The C2C12 cells treated under different treatment conditions were seeded in 24-well plates at a rate of 1 × 10^5^ cells / well and incubated for 24h. The 5-erhynyl-20-deoxyuridine (EDU) incorporation assay was performed using an EDU assay kit (#COO75S, Beyotime Biotechnology) according to the manufacturer’s instructions. The proportion of EDU-positive cells was counted using a laser scanning confocal microscope (Olympus) [72–74]. Similar to our previous research, Cell proliferation was detected with TransDect CCK (TransGen Biotech, Beijing, China) according to the manufacturer’s protocol after transfection.

### Gene set enrichment analysis (GSEA)

GSEA is a "molecular signature database" used to investigate potential mechanisms using the project of JAVA (http://software.broadinstitute.org/gsea/index.jsp) [75]. The number of random samples was set to 1000, and the threshold for statistical significance was set at P < 0.05.

### Statistical analysis

Statistical analysis was performed using GraphPad Prism (version 7.0) software. Results are expressed as the mean ± standard deviation of three or six independent experiments. Statistical significance between groups was determined using one-way analysis of variance or two-tailed t-test. Correlation analysis was performed using Pearson’s correlation. *P <0.05 was considered to be statistically significant.

### Ethical statement

This study was approved by the Institutional Ethics Review Board of Shanghai General Hospital, Shanghai Jiao Tong University, Shanghai, China.

### Informed Consent

Written informed consent was sought from all the enrolled subjects.

### Availability of data and materials

Data pertaining to this study is available from corresponding author upon reasonable request.

## Supplementary Material

Supplementary Tables

Supplementary Figure 1
