# Supplementary material for: lncRNA DLEU2 acts as a miR-181a sponge to regulate SEPP1 and inhibit skeletal muscle differentiation and regeneration
Source: Aging (Albany NY). 2020 Nov 18;12(23):24033–56. doi: 10.18632/aging.104095 (PMC7762514; doi:10.18632/aging.104095)
Supplement: Supplementary Tables [file aging-12-104095-s001.pdf]

SUPPLEMENTARY TABLES

Supplementary Table 1. Prediction factors for nomogram.

Variable	Prediction model		
	**Coef**	**The regression coefficient (95% CI)**	**P−value**
Intercept	2.08	0.79 to 3.56	0.0031
SEPP1	1.74	0.39 to 3.35	0.0174
CFOD1	-2.62	−4.23 to −1.30	0.0003
GOT1	-1.78	−3.12 to −0.52	0.0072
SV2A	-1.54	−2.92 to −0.30	0.0189

Note: Coef is the regression coefficient; Total n=92; Null Deviance:128.9; Residual Deviance:65.27; AIC: 75.27

Supplementary Table 2.The proposed nomogram of accuracy, F-value, precision and recall.

		Predicted class		
		**Positive**	**Negative**	**Total**
Concrete class	TURE	TP	FN	P
	FALSE	FP	TN	N
	Total	P'	N'	P+N

The calculation formulas:	
Accuracy	ACC=(TP+TN)/(TP+TN+FP+FN)
F-value(α=1)	F1=2TP/(2TP+FP+FN)
Precision	P=TP/(TP+FP)
Recall	Recall=TP/(TP+FN)
