# Supplementary material for: lncRNA DLEU2 acts as a miR-181a sponge to regulate SEPP1 and inhibit skeletal muscle differentiation and regeneration
Source: Aging (Albany NY). 2020 Nov 18;12(23):24033–56. doi: 10.18632/aging.104095 (PMC7762514; doi:10.18632/aging.104095)
Supplement: Supplementary Figure 1 [file aging-12-104095-s002.pdf]

SUPPLEMENTARY FIGURE

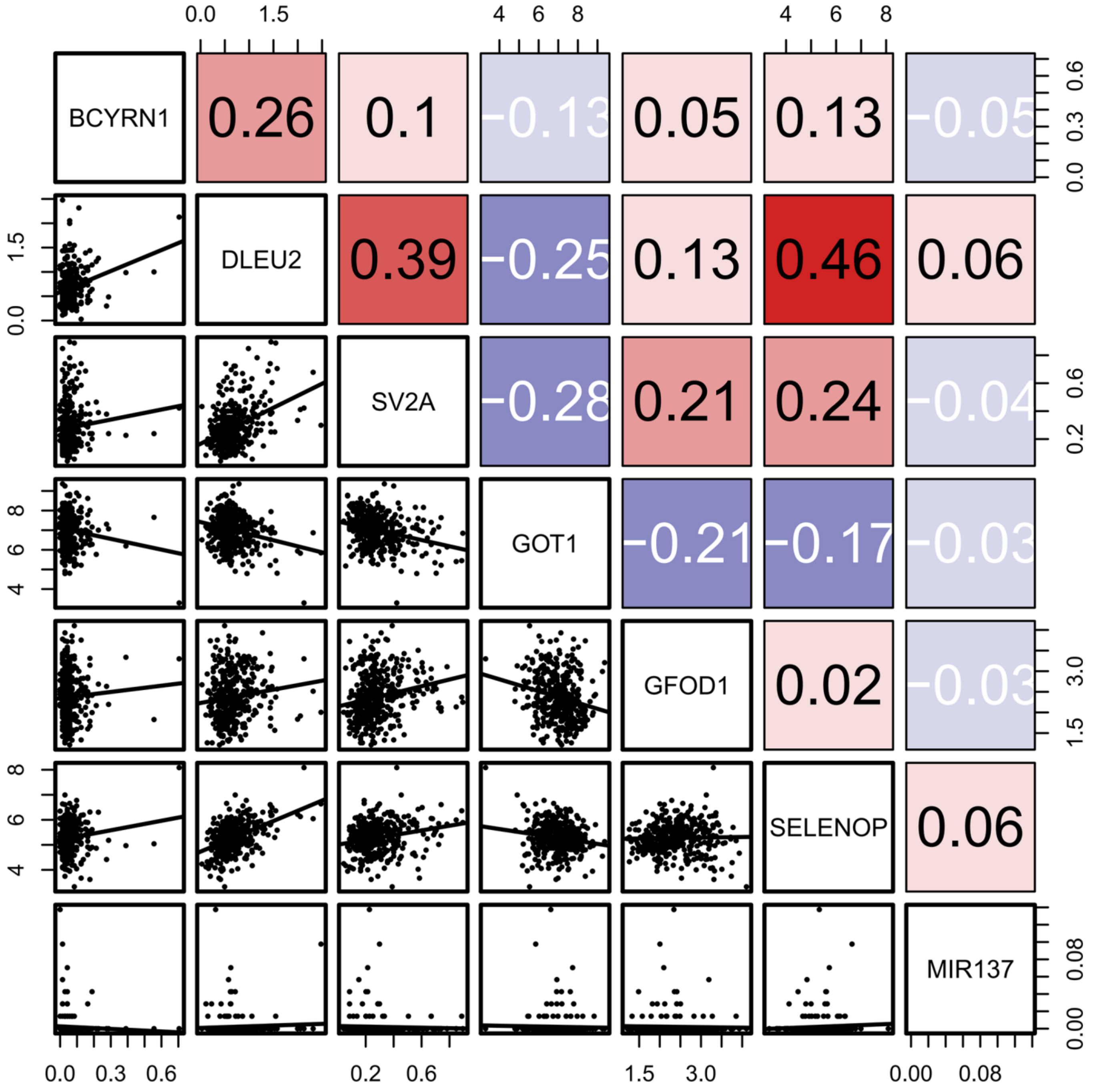

Supplementary Figure 1. Correlations among BCYRN1, DLEU2, SV2A, GOT1, GFOD1, SELENOP (SEPP1) and miR-137. The correlations shown are for muscle tissues from GTEX cohorts (n=396).
